# Hyperspectral Reflectance Imaging Technique for Visualization of Moisture Distribution in Cooked Chicken Breast

**DOI:** 10.3390/s131013289

**Published:** 2013-09-30

**Authors:** Lalit Mohan Kandpal, Hoonsoo Lee, Moon S. Kim, Changyeun Mo, Byoung-Kwan Cho

**Affiliations:** 1 Department of Biosystems Machinery Engineering, College of Agricultural and Life Science, Chungnam National University, 99 Daehak-ro, Yuseong-gu, Daejeon 305-764, Korea; E-Mails: lalitm85@gmail.com (L.M.K.); hoonsoolee83@gmail.com (H.L.); 2 Environmental Microbiology and Food Safety Laboratory, Agricultural Research Service, US Department of Agriculture, Powder Mill Rd. Bldg. 303, BARC-East, Beltsville, MD 20705, USA; E-Mail: moon.kim@ars.usda.gov; 3 National Academy of Agricultural Science, Rural Development Administration, 88-2 Seodun-dong, Gwonseon-gu, Suwon, Gyeonggi-do 441-100, Korea; E-Mail: cymoh100@korea.kr

**Keywords:** hyperspectral imaging, chicken breast, moisture content, cooking oven, PLSR

## Abstract

Spectroscopy has proven to be an efficient tool for measuring the properties of meat. In this article, hyperspectral imaging (HSI) techniques are used to determine the moisture content in cooked chicken breast over the VIS/NIR (400–1,000 nm) spectral range. Moisture measurements were performed using an oven drying method. A partial least squares regression (PLSR) model was developed to extract a relationship between the HSI spectra and the moisture content. In the full wavelength range, the PLSR model possessed a maximum R^2^p of 0.90 and an SEP of 0.74%. For the NIR range, the PLSR model yielded an R^2^p of 0.94 and an SEP of 0.71%. The majority of the absorption peaks occurred around 760 and 970 nm, representing the water content in the samples. Finally, PLSR images were constructed to visualize the dehydration and water distribution within different sample regions. The high correlation coefficient and low prediction error from the PLSR analysis validates that HSI is an effective tool for visualizing the chemical properties of meat.

## 1. Introduction

Chicken meat is preferred across the World due to its high nutrient value (protein content) and flavor. Consumers continually demand high meat quality. In order to impart high quality and flavor, proper cooking methods, including optimal cooking time, temperature, and handling, are essential.

In practice, new research is often developed to increase the quality of meat in order to fulfill consumer needs. Meat quality is determined by its chemical and physical properties. A change in these properties influences juiciness, texture, fat composition, water content (moisture), size and other properties. These properties generally change during different processing techniques such as thawing, chilling, marinating, handling and cooking. In particular, moisture plays an important role in creating a juicy texture and taste.

Cooking is the prime factor impacting the texture and the toughness of meat. During the cooking process, structural and physical changes occur, such as protein-denaturation and coagulation. At 65 °C, moisture loss occurs. The muscles in raw poultry meat are composed of 75% water, 65% of which remains after cooking due to evaporation and fluid loss. Water molecules in food products are generally influenced by environmental changes such as thermal processing, pH, light, and pressure. These factors affect the molecular state of water, the component reactivity, and the functional characteristics. For instance, heat causes both dehydration and physiochemical changes, such as protein denaturation in meat samples [1,2]. The change in meat quality during cooking is known as cooking loss, during which a loss of fluid causes a change in the nutritional content. Changes in shape, moisture, weight, and texture can also occur, suggesting that temperature strongly influences these parameters.

In general, a higher cooking temperature results in a greater moisture loss. Therefore, it is necessary to determine the best temperature and time for cooking meat products. Overcooking can cause additional moisture loss, while undercooking leaves the meat raw. Several studies have been conducted on quality measurement of raw meats using nondestructive methods [3–5]; however, there are few approaches for nondestructive quality measurements of cooked meats [6]. If the quality of cooked meats can be measured quantitatively, the cooking process can be controlled precisely.

An instrumental method, such as hyperspectral imaging, in combination with a multivariate data analysis technique, such as partial least squares regression, can provide reliable information on visualizing quality in meats such as beef, pork, chicken, and fish [7,8]. Near infrared (NIR) hyperspectral imaging is a rapid, non-destructive, and accurate technique that can provide useful measurements of meat properties without requiring sample preparation. Hyperspectral imaging (HSI) is a three-dimensional technique, consisting of both images and spectra, which yields physical and chemical information. These hyperspectral techniques are the best potential tool for measuring the water content and distribution in the meat samples [9]. This information cannot be obtained using any other conventional technique. For this reason, we apply hyperspectral imaging techniques to determine the moisture content in cooked chicken breast meat with the following objectives: (a) Establish a visual/near infrared (VIS/NIR) hyperspectral imaging system (400–1,000 nm) for obtaining spectrum of the cooked chicken breast samples. (b) Develop a PLSR model to analyze the spectral data among the measured moisture values and identify the effective wavelength related to the meat water content. (c) Develop image processing algorithms for the visualization of chemical images of the moisture at three different temperature variations.

## 2. Experimental Section

### 2.1. Sample Preparation

Chicken breast samples were obtained from a local market in South Korea. A total of 36 raw chicken breasts were used in this study. Before cooking, samples were stored for 24 h at 4 °C in the laboratory. The samples were cooked in an oven (LG Electronics, Changwon, Korea) at three different temperatures, 50 °C, 60 °C, and 70 °C, with 12 samples at each temperature. The temperature of the chicken breasts was measured in real time using a thermometer (K-type, TES-1311A, TES Electronic Corp., Taipei, Taiwan) placed inside the samples. After being cooked, two slices were obtained from the central and peripheral parts of each chicken breast, as we assumed that the moisture content could vary in different parts of the meat. Hence, a total 72 samples were prepared for investigation. Each sample was scanned with a hyperspectral imaging system. The moisture content was extracted using the laboratory method described below.

### 2.2. Moisture Measurement

The total moisture content in the chicken breast samples was determined using the oven drying method, as described by the Association of Official Analytical Chemists (AOAC). Thus, the moisture content of each sample was calculated as a proportion of the weight loss after oven drying. The original weights of the samples were measured with pre-weighed aluminum pans, and the samples were dried in a vacuum oven for 24 h. The dried sample weights were again measured with the aluminum pans, and the percentage loss in moisture content was measured with the following equation:

(1) $$\text{Moisture content}(\%)=({\text{W}}_{2}-{\text{W}}_{3})/({\text{W}}_{2}-{\text{W}}_{1})\times 100$$

where W_1_ is the weight of the dry aluminum pan, W_2_ is the weight of the wet sample and the dry aluminum pan, and W_3_ is the weight of the dry sample and the dry aluminum pan.

### 2.3. Hyperspectral Imaging System

**Figure 1.**
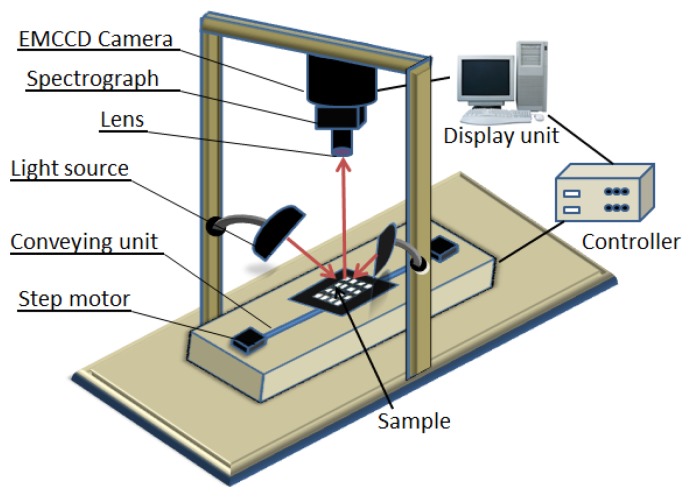
Hyperspectral imaging system.

The VIS/NIR line scan hyperspectral imaging system, shown in Figure 1, was used to obtain spectral images at reflectance modes of the samples in the 400–1,000 nm wavelength range. The HSI system is composed of an EMCCD camera (Luca RDL-604M, Andor Technology, South Windsor, CT, USA), and a line scan imaging spectrograph (VIS/NIR, Headwall Photonics, Fitchburg, MA, USA) with the spectral wavelength range of 400–1,000 nm (VIS/NIR).

### 2.4. Image Acquisition and Correction

Chicken samples were placed in the sample holder to restrict the sample displacement and transported to a conveyor unit. The conveyor speed, field of view (FOV), motor speed, exposure time, and wavelength were controlled using Visual Basic software. When the sample moved within the FOV, the images and spectra were acquired by the EMCCD camera and sent to a computer for storage and post processing. The acquired hyperspectral images were stored as a hyper-cube consisting of two spatial dimensions (x and y) and one spectral wavelength dimension (λ) from which both the physical and the chemical properties of the samples could be obtained.

The dark current in the camera led to noise in the sample images. In order to correct the noise caused by the camera, both white and dark reference images were acquired. The white image (W) was obtained using a white Teflon material with a 99% reflectance, while the dark image (D) was acquired by covering the lens using the lens cover with a 0% reflectance. These reference images were used to calculate the reflectance value of the raw spectral image (R_0_) of the sample using the following equation:

(2) $$\text{R}=({\text{R}}_{0}-{\text{D}}_{\text{i}})/({\text{W}}_{\text{i}}-{\text{D}}_{\text{i}})$$

where R_0_ is the raw hyperspectral image, W is the reflectance image, D is the dark image, i is the pixel index (i = 1,2,3,…,n), and n is the total number of pixels.

### 2.5. Image Separation

**Figure 2.**
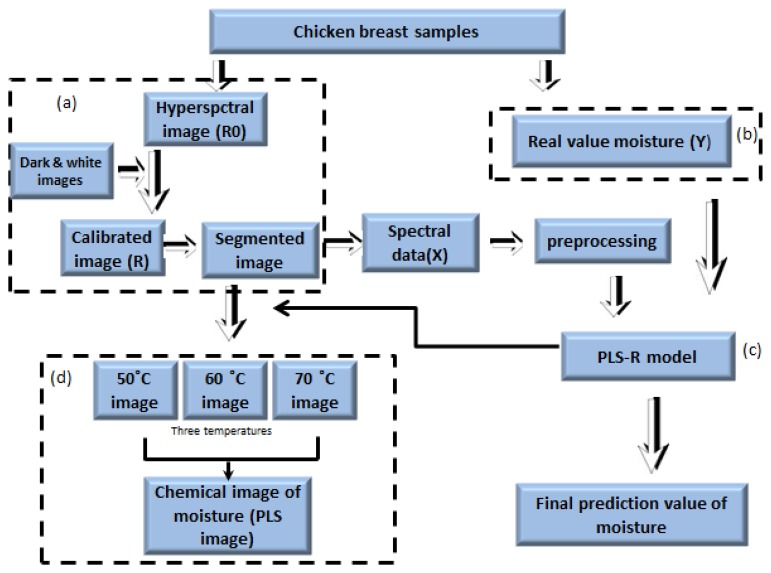
Experiments and image processing flow: (**a**) acquiring and correcting spectral images; (**b**) measurement of moisture contents; (**c**) prediction of moisture content using a PLSR model and (**d**) PLSR images of the moisture content for different temperatures.

We used image separation to remove the background from the obtained hyperspectral images. The background was removed by identifying a threshold value after subtracting the low reflectance image value from the high reflectance image value. Figure 2a shows the steps of image correction of the entire image processing.

### 2.6. Spectrum Calibration and Data Analysis

#### 2.6.1. Data Preprocessing and Multivariate Data Analysis

The raw data extracted from the hyperspectral imaging contained scattering noise generated from the camera. In order to overcome the scattering interference, we applied seven mathematical pre-treatments, such as normalization, standard normal variate (SNV) transformation, multiple scatter correction (MSC) and Savitzky-Golay filtering. The purpose of SNV is to remove the additive baseline and the multiplicative signal effects, transforming the spectrum with zero mean and variance equal to one. The aim of MSC is to remove the effect of physical light scatter from the spectrum (compensation for particle size effects). Finally, the Savitzky-Golay filter is a moving window method, which separates the overlapped peaks from the spectra.

A multivariate data analysis method, partial least square regression, was used to establish quantitative models between the preprocessed spectral data and the measured reference values of moisture content. The regression model predicts the sample property from the spectral data. PLSR is a commonly used statistical tool for spectral data processing and developing a predictive model. This model is capable of processing a large amount of spectral data and provides a quantitative relationship. The PLSR equations are given by:

(3) $$\text{X}={\text{TP}}^{\text{T}}+\text{E}$$

(4) $$\text{Y}={\text{UQ}}^{\text{T}}+\text{E}$$

where the spectral data matrix X is decomposed into the score matrix T, loading matrix P, and error matrix E. Likewise, the reference values matrix Y is decomposed into the score matrix U, loading matrix Q, and error matrix E.

In PLSR, the data is compressed into orthogonal structures called latent variables (LV) or latent factors [10]. Further, LVs describe the maximum covariance between the spectral data and the response variables (e.g., the measured moisture contents in this study). The optimal number of LVs was determined by the lowest value of the predicted root mean square error (RMSE) values using the following equation:

(5) $$\text{RMSE}=\sqrt{\frac{1}{z}\sum_{i=1}^{z}{\left(y_{i}-{\hat{y}}_{i}\right)}^{2}}$$

where y_i_ is the true reference value, *ŷ*_i_ is the predicted value from the PLSR, and z is the number of predictions. The coefficient of determination (*R*^2^) yields the prediction ability of the model. We used Matlab software (The Math Works, Natick, MA, USA) for the PLSR analysis.

#### 2.6.2. Data Partition

The spectral data for all of the samples was arranged in a matrix (X) whose rows represent the number of samples and whose columns represent the number of variables (wavelengths). The measured reference values were arranged in the matrix Y, where the columns of Y hold the measured reference values of moisture content obtained by the oven drying method. The whole dataset (72 samples) was split into two groups: a training group consisting of 52 samples and a testing group consisting of 20 samples. The PLSR models were built with the training group using a full cross-validation method (leave-one-out;, removing one observation at one time from the sample sets until all samples have been removed once. During the cross-validation, only the first few LVs are required because they contain the most variation. However, using too many LVs may cause over-fitting in the data. We estimated the correct number of LVs using the RMSE method and the performance of the PLSR model reported as the acquired accuracy of *R*^2^ (coefficient of determination). The best model had a high coefficient of determination and low standard errors. Finally, the model was validated with the testing group (20 samples).

#### 2.6.3. PLSR Image Processing

**Figure 3.**
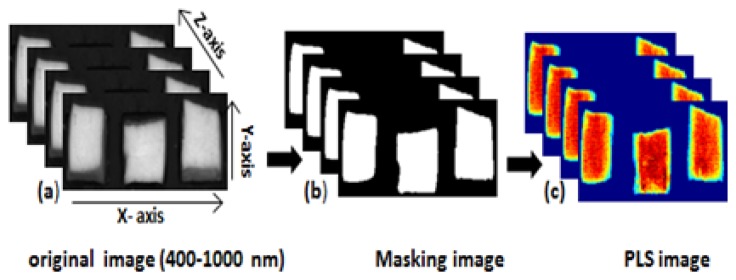
Image processing steps for generating PLS images: (**a**) Original image; (**b**) Masking image and (**c**) PLS image.

To obtain a moisture concentration map for the samples, a PLSR image was generated by multiplying the beta coefficient from the PLSR model with the spectrum of each pixel in the image. In order to remove the background, a masking image was generated for the PLSR images as mentioned in Figure 3. The PLSR image algorithm is given by:

(6) $${\text{PLS}}_{\text{image}}=\sum_{i=1}^{n}\beta i*Hi+\text{Constant}$$

## 3. Results and Discussion

### 3.1. Spectral Features of Chicken Breast Samples

**Figure 4.**
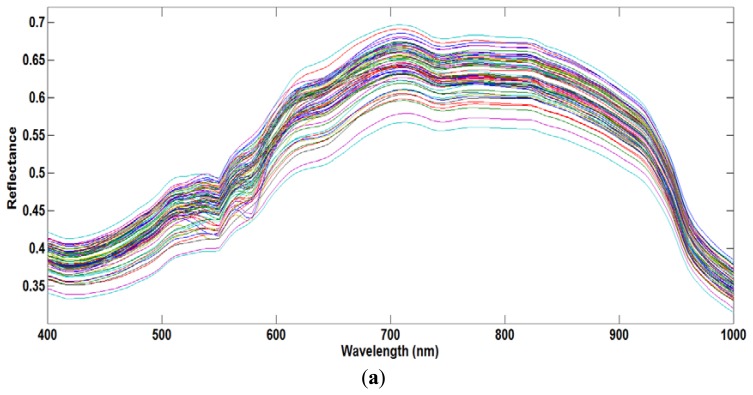

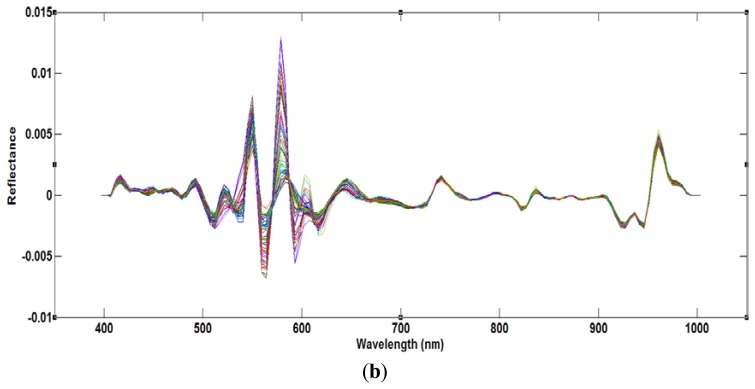
(**a**) Raw spcectra of chicken samples; (**b**) Second derivative preprocessed spectra of chicken samples.

The moisture content, measured using the oven drying method, ranged from 66% to 73%. Figure 4a shows the average raw spectra of the 72 samples acquired from the VIS/NIR system in the spectral range of 400–1,000 nm, and Figure 4b shows the resulting preprocessed spectral profiles. The NIR region is related to several broadband peaks and fundamental vibrations in the meat samples, such as C-H, O-H and O-H functional groups [7]. At first, the PLSR model was developed using the full VIS/NIR spectral region. Using the beta coefficient curve, we assumed that the O-H functional group related to the moisture content falls in the NIR region. Therefore, to reduce inessential information, a second model was developed covering only the NIR region. Previous studies [7,11] showed that the effective wavelength region has a higher model accuracy compared to the full wavelength.

### 3.2. Multivariate Data Analysis

#### 3.2.1. PLSR Results Based on Full Wavelength (VIS/NIR)

**Figure 5.**
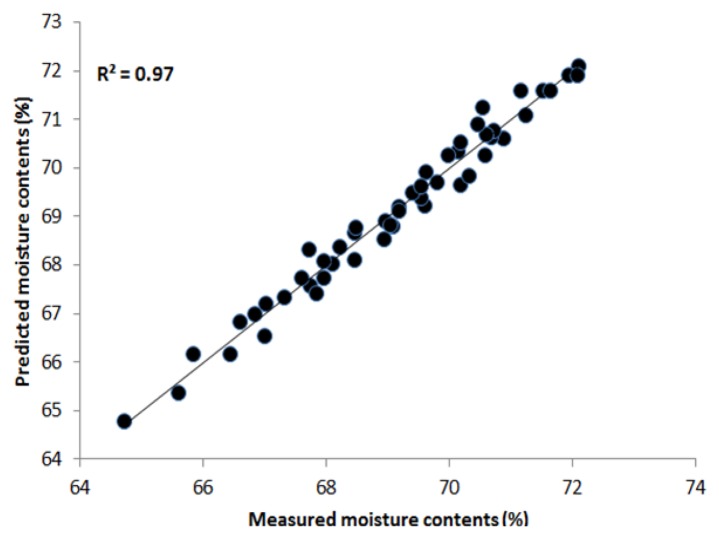
Regression plot of measured and predicted moisture data (400–1,000 nm).

**Figure 6.**
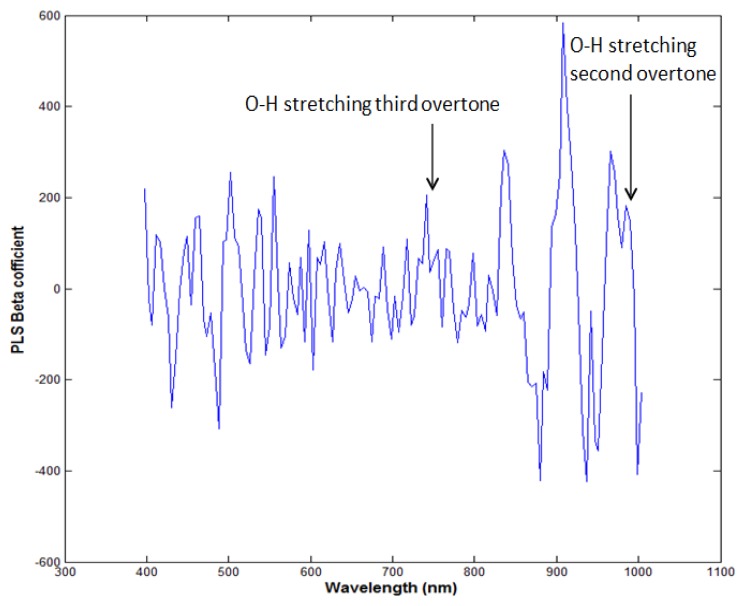
Beta coefficient plot of moisture content with PLSR model in the range of 400–1,000 nm.

PLSR was applied to the processed data using the entire wavelength range as a predictor. The data was preprocessed by seven pre-processing methods. Table 1 shows that the highest acquired correlation coefficient values from the SNV pre-processing was R^2^ c = 0.97 (calibration) and R^2^ p = 0.90 (prediction). The standard error of calibration (SEC) and standard error of prediction (SEP) were 0.27% and 0.74%, respectively. The optimal number of latent variables was determined from minimum RMSE values in the cross-validation process. Figure 5 shows the measured moisture reference values and the values predicted by the PLSR model for the chicken samples. The predicted values for moisture content (g/100 g) range from 64% to 73%, and we see that there is a strong correlation between the measured and predicted values. The beta coefficient graph in Figure 6 shows the broadband peaks in the spectrum. Most absorption peaks appeared around 760 nm (weak band) and 970 nm, which are related to the third and second overtones of the O-H stretching modes, representing the water content in the samples [7].

#### 3.2.2. PLSR Results Based on NIR Region

**Figure 7.**
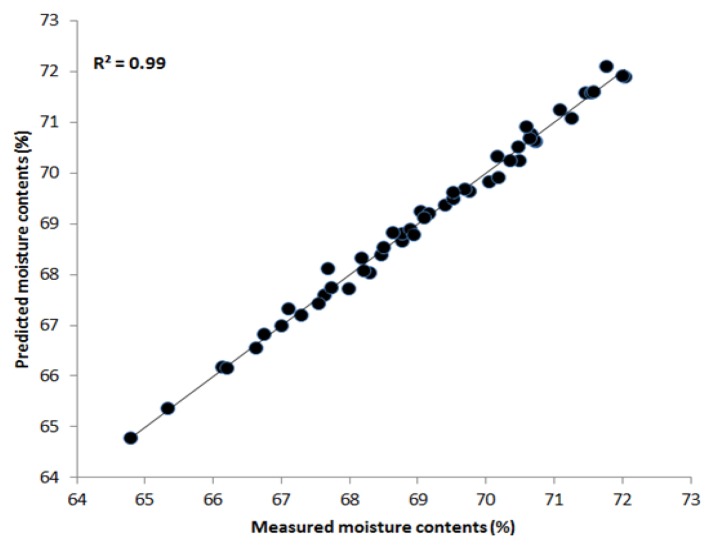
Regression plot of raw and predicted moisture data (700–1,000 nm).

We applied similar PLSR multivariate techniques and preprocessing methods to estimate the moisture content of the chicken samples in the NIR region between 700 nm and 1,000 nm. Table 2 demonstrates that the maximum correlation coefficient values obtained by the Savitzky-Golay second derivative of R^2^ c and R^2^ p were 0.99 and 0.94, respectively. The SEC and SEP values were 0.15% and 0.71%, respectively. Figure 7 shows the prediction plot of moisture content where most of the points fall upon the regression line. These results suggest that the effective NIR region of 700–1,000 nm is better for predicting water content in the samples and for convenience during the analysis. Hence, HSI in combination with PLSR is an effective tool for determining chemical features in meat.

#### 3.2.3. Appearance of Chicken Samples during Different Temperatures

**Figure 8.**
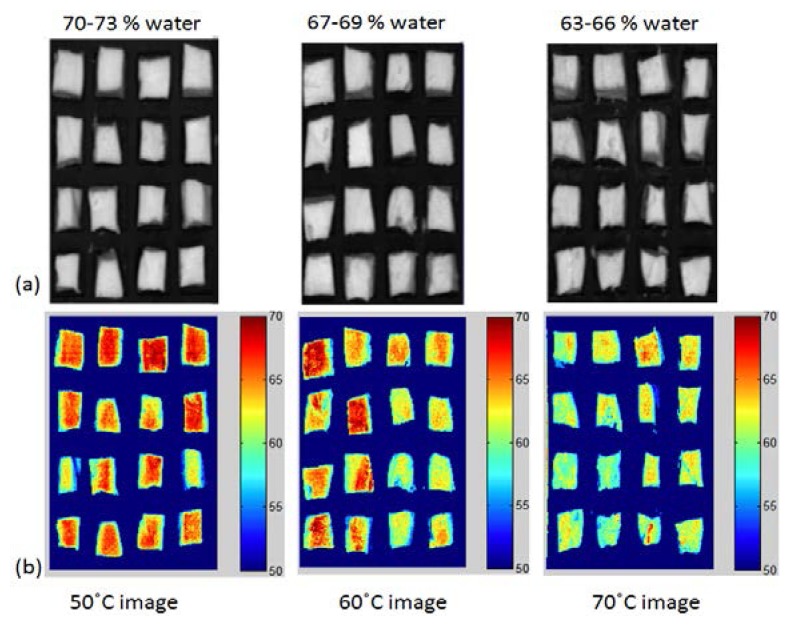
(**a**) Original hyperspectral images; (**b**) Chemical images of moisture images.

Figure 8 shows the final PLSR images of the moisture content of the chicken breast meat cooked at different temperatures. The images were constructed by multiplying the obtained beta coefficient (regression coefficient) from the PLSR model with the spectra of each pixel in the image. This image represents a water dispersion map in the chicken samples. The water content is higher in the central part of the sample than in the peripheral part. This observation is due to the higher contact with hot air around the peripheral part. Samples cooked at 50 °C contained 70%–73% water, while the 70 °C samples had a water content of 63%–66%. The difference between the samples is highly enhanced in the RGB images, shown in Figure 8b. As the temperature increased, a higher water loss occurred, which is indicated by the disappearance of the red pixels in the 70 °C images. This dehydration process from region to region cannot be visualized by any other conventional imaging technique. Therefore, HSI combined with image processing techniques can be used to clearly detect chemical changes in meat samples.

## 4. Conclusions

The present study utilizes VIS/NIR hyperspectroscopy imaging techniques for predicting and visualizing the moisture content of cooked chicken meat samples. The obtained moisture content extracted from the instrument suggests that our method is rapid, non-destructive, and highly sensitive to the moisture content and the changing properties of water in the meat. The PLSR results revealed good performance and a high correlation for predicting moisture content in the samples when using the full wavelength (R^2^ p= 0.90) and effective NIR region (R^2^ p = 0.94) spectral data and measured reference values. In addition, the PLSR beta coefficient plot provides the broadband peaks for the O-H functional group present in the samples. When used in combination with PLSR, HSI techniques are capable of visualizing the changing properties of water in meat, which is not possible by other methods. The overall results suggested that HSI techniques are suitable for estimating and visualizing the chemical features (moisture, protein and fat contents) of meat. For our future work, we plan to employ SWIR hyperspectral imaging (1,100–2,500 nm) on various meat products to detect different chemical properties.

## Figures and Tables

**Table 1.** PLS results with full spectral region (400–1,000 nm).

**Parameter**	**Preprocessing**	**Factors**	**Calibration**		**Validation**		**Prediction**	

			**R^2^c**	^d^**SEC (%)**	**R^2^v**	^d^**SEV (%)**	**R^2^p**	^d^**SEP (%)**
Moisture contents	Mean Normilization	19	0.97	0.26	0.87	0.63	0.89	0.71
	Max Normilization	19	0.97	0.25	0.87	0.63	0.89	0.75
	Range Normilization	19	0.97	0.26	0.87	0.64	0.89	0.73
	^a^ SNV	18	0.97	0.27	0.86	0.66	0.90	0.74
	^b^ MSC	18	0.98	0.25	0.88	0.61	0.89	0.79
	^c^ First Derivative	16	0.96	0.31	0.85	0.71	0.58	2.75
	^c^ Second Derivative	10	0.96	0.35	0.78	0.83	0.78	1.04
	Raw	18	0.97	0.30	0.85	0.68	0.89	0.71

Notes:

a Standard Normal Variate;

b Multiple Scatter Correction;

c Savitzky-Golay First and Second Derivatives;

d SEC, ^d^ SEV and ^d^ SEP are the standard error of calibration, validation and prediction;

e *R*^2^ is the correlation coefficient.

**Table 2.** PLS results with NIR region (700–1,000 nm).

**Parameter**	**Preprocessing**	**Factors**	**Calibration**		**Validation**		**Prediction**	

			**R^2^c**	^d^**SEC (%)**	**R^2^v**	^d^**SEV (%)**	**R^2^p**	^d^**SEP (%)**
Moisture contents	Mean Normilization	13	0.98	0.20	0.89	0.57	0.93	0.61
	Max Normilization	15	0.99	0.16	0.89	0.59	0.93	0.62
	Range Normilization	18	0.99	0.11	0.88	0.61	0.92	0.68
	^a^ SNV	17	0.99	0.12	0.90	0.57	0.91	0.77
	^b^ MSC	13	0.98	0.19	0.89	0.58	0.93	0.58
	^c^ First Derivative	14	0.97	0.26	0.88	0.59	0.58	5.39
	^c^ Second Derivative	13	0.99	0.15	0.90	0.55	0.94	0.71
	Raw	15	0.98	0.19	0.89	0.58	0.93	0.61

Notes:

a Standard Normal Variate;

b Multiple Scatter Correction;

c Savitzky-Golay First and Second Derivatives;

d SEC, ^d^ SEV and ^d^ SEP are the standard error of calibration, validation and prediction;

e *R*^2^ is the correlation coefficient.
